# Supplementary material for: Logistic Regression Algorithm Differentiates Gulf War Illness (GWI) Functional Magnetic Resonance Imaging (fMRI) Data from a Sedentary Control
Source: Brain Sci. 2020 May 25;10(5):319. doi: 10.3390/brainsci10050319 (PMC7287630; doi:10.3390/brainsci10050319)
Supplement: Supplementary file 1 [file brainsci-10-00319-s001.pdf]

Supplemental Data:
SOM. Table S1. AAL Atlas centers of mass (MNI coordinates) and voxels per region.
https://figshare.com/articles/_Abbreviations_and_MNI_coordinates_of_AAL_/184981

Index	L R	Abbreviation	X	Y	Z	Voxels
1	L	Precentral_L	−39	−6	51	3526
2	R	Precentral_R	41	−8	52	3381
3	L	Frontal_Sup_L	−18	35	42	3599
4	R	Frontal_Sup_R	22	31	44	4056
5	L	Frontal_Sup_Orb_L	−17	47	−13	963
6	R	Frontal_Sup_Orb_R	18	48	−14	997
7	L	Frontal_Mid_L	−33	33	35	4863
8	R	Frontal_Mid_R	38	33	34	5104
9	L	Frontal_Mid_Orb_L	−31	50	−10	888
10	R	Frontal_Mid_Orb_R	33	53	−11	1015
11	L	Frontal_Inf_Oper_L	−48	13	19	1038
12	R	Frontal_Inf_Oper_R	50	15	21	1399
13	L	Frontal_Inf_Tri_L	−46	30	14	2529
14	R	Frontal_Inf_Tri_R	50	30	14	2151
15	L	Frontal_Inf_Orb_L	−36	31	−12	1690
16	R	Frontal_Inf_Orb_R	41	32	−12	1707
17	L	Rolandic_Oper_L	−47	−8	14	990
18	R	Rolandic_Oper_R	53	−6	15	1331
19	L	Supp_Motor_Area_L	−5	5	61	2147
20	R	Supp_Motor_Area_R	9	0	62	2371
21	L	Olfactory_L	−8	15	−11	280
22	R	Olfactory_R	10	16	−11	289

23	L	Frontal_Sup_Medial_L	−5	49	31	2992
24	R	Frontal_Sup_Medial_R	9	51	30	2134
25	L	Frontal_Med_Orb_L	−5	54	−7	719
26	R	Frontal_Med_Orb_R	8	52	−7	856
27	L	Rectus_L	−5	37	−18	852
28	R	Rectus_R	8	36	−18	745
29	L	Insula_L	−35	7	3	1858
30	R	Insula_R	39	6	2	1770
31	L	Cingulum_Ant_L	−4	35	14	1400
32	R	Cingulum_Ant_R	8	37	16	1313
33	L	Cingulum_Mid_L	−5	−15	42	1941
34	R	Cingulum_Mid_R	8	−9	40	2203
35	L	Cingulum_Post_L	−5	−43	25	463
36	R	Cingulum_Post_R	7	−42	22	335
37	L	Hippocampus_L	−25	−21	−10	932
38	R	Hippocampus_R	29	−20	−10	946
39	L	ParaHippocampal_L	−21	−16	−21	978
40	R	ParaHippocampal_R	25	−15	−20	1132
41	L	Amygdala_L	−23	−1	−17	220
42	R	Amygdala_R	27	1	−18	248
43	L	Calcarine_L	−7	−79	6	2258
44	R	Calcarine_R	16	−73	9	1861
45	L	Cuneus_L	−6	−80	27	1526
46	R	Cuneus_R	14	−79	28	1424
47	L	Lingual_L	−15	−68	−5	2095

48	R	Lingual_R	16	−67	−4	2300
49	L	Occipital_Sup_L	−17	−84	28	1366
50	R	Occipital_Sup_R	24	−81	31	1413
51	L	Occipital_Mid_L	−32	−81	16	3270
52	R	Occipital_Mid_R	37	−80	19	2098
53	L	Occipital_Inf_L	−36	−78	−8	941
54	R	Occipital_Inf_R	38	−82	−8	989
55	L	Fusiform_L	−31	−40	−20	2310
56	R	Fusiform_R	34	−39	−20	2518
57	L	Postcentral_L	−42	−23	49	3892
58	R	Postcentral_R	41	−25	53	3823
59	L	Parietal_Sup_L	−23	−60	59	2065
60	R	Parietal_Sup_R	26	−59	62	2222
61	L	Parietal_Inf_L	−43	−46	47	2447
62	R	Parietal_Inf_R	46	−46	50	1345
63	L	SupraMarginal_L	−56	−34	30	1256
64	R	SupraMarginal_R	58	−32	34	1974
65	L	Angular_L	−44	−61	36	1173
66	R	Angular_R	46	−60	39	1752
67	L	Precuneus_L	−7	−56	48	3528
68	R	Precuneus_R	10	−56	44	3265
69	L	Paracentral_Lobule_L	−8	−25	70	1349
70	R	Paracentral_Lobule_R	7	−32	68	836
71	L	Caudate_L	−11	11	9	962
72	R	Caudate_R	15	12	9	994

73	L	Putamen_L	−24	4	2	1009
74	R	Putamen_R	28	5	2	1064
75	L	Pallidum_L	−18	0	0	293
76	R	Pallidum_R	21	0	0	280
77	L	Thalamus_L	−11	−18	8	1100
78	R	Thalamus_R	13	−18	8	1057
79	L	Heschl_L	−42	−19	10	225
80	R	Heschl_R	46	−17	10	249
81	L	Temporal_Sup_L	−53	−21	7	2296
82	R	Temporal_Sup_R	58	−22	7	3141
83	L	Temporal_Pole_Sup_L	−40	15	−20	1285
84	R	Temporal_Pole_Sup_R	48	15	−17	1338
85	L	Temporal_Mid_L	−56	−34	−2	4942
86	R	Temporal_Mid_R	57	−37	−1	4409
87	L	Temporal_Pole_Mid_L	−36	15	−34	755
88	R	Temporal_Pole_Mid_R	44	15	−32	1187
89	L	Temporal_Inf_L	−50	−28	−23	3200
90	R	Temporal_Inf_R	54	−31	−22	3557
91	L	Cerebellum_Crus1_L	−35	−67	−29	2603
92	R	Cerebellum_Crus1_R	38	−67	−30	2648
93	L	Cerebellum_Crus2_L	−28	−73	−38	1894
94	R	Cerebellum_Crus2_R	33	−69	−40	2117
95	L	Cerebellum_3_L	−8	−37	−19	136
96	R	Cerebellum_3_R	13	−34	−19	207
97	L	Cerebellum_4_5_L	−14	−43	−17	1125

98	R	Cerebellum_4_5_R	18	−43	−18	861
99	L	Cerebellum_6_L	−22	−59	−22	1694
100	R	Cerebellum_6_R	26	−58	−24	1795
101	L	Cerebellum_7b_L	−31	−60	−45	585
102	R	Cerebellum_7b_R	34	−63	−48	534
103	L	Cerebellum_8_L	−25	−55	−48	1887
104	R	Cerebellum_8_R	26	−56	−49	2308
105	L	Cerebellum_9_L	−10	−49	−46	869
106	R	Cerebellum_9_R	10	−49	−46	809
107	L	Cerebellum_10_L	−22	−34	−42	144
108	R	Cerebellum_10_R	27	−34	−41	159
109	midline	Vermis_1_2	2	−39	−20	53
110	midline	Vermis_3	2	−40	−11	228
111	midline	Vermis_4_5	2	−52	−6	665
112	midline	Vermis_6	2	−67	−15	371
113	midline	Vermis_7	2	−72	−25	194
114	midline	Vermis_8	2	−64	−34	243
115	midline	Vermis_9	2	−55	−35	174
116	midline	Vermis_10	1	−46	−32	112

Figure S1. AAL atlas depicting all regions on sedentary control brain. Figure generated in Nilearn python package.

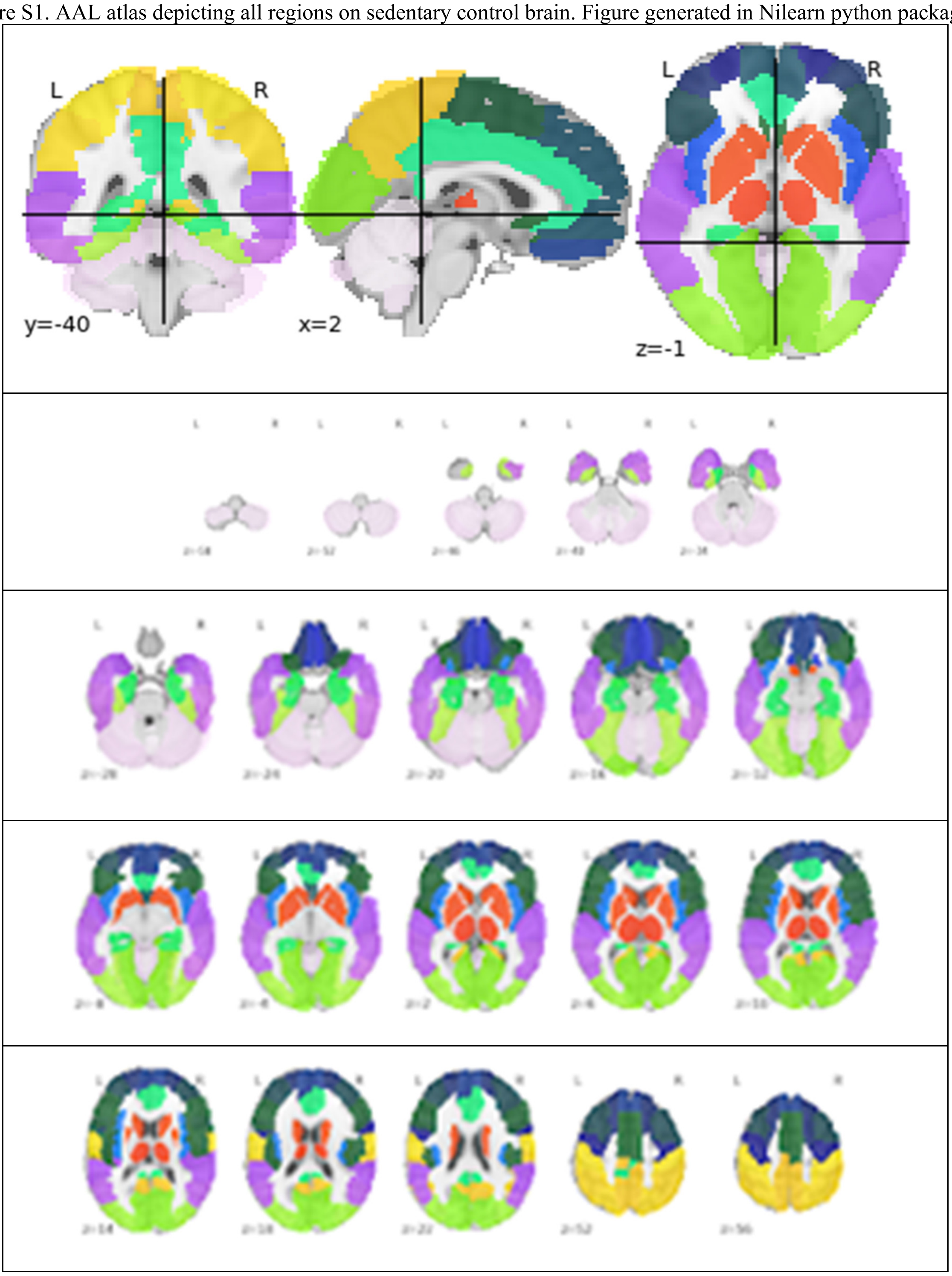

Table S2. Initial testing data depicting model results for preliminary model runs for alternate techniques and reasoning behind selection of logistic.

Method	Average on Repeated Runs - Day 1	Average on Repeated Runs - Day 2	Ease of Deployment	Time to Run
Nearest Neighbors	71.30%	78.70%	Simple Code	0.23 s
Linear SVM	59.26%	81.48%	Simple Code	0.13 s
Decision Tree	66.67%	84.26%	Simple Code	0.20 s
Random Forest	72.22%	82.41%	Complicated	9.40 s
AdaBoost	68.52%	83.33%	Complicated	3.34 s
Naive Bayes	70.37%	84.07%	Simple Code	0.54 s
QDA	72.22%	77.78%	Complicated	15.43 s
*** Logistic Regression**	**70.60%**	**85.30%**	**Simple Code**	**0.10 s**
Multi-layer Perceptron (MLP) - Neural Net	59.26%	80.56%	Complicated	105.43 s
Support Vector Machine	0.6666666666666666	0.6851851851851852	Complicated	24 hr+
